# Study protocol of an equivalence randomized controlled trial to evaluate the effectiveness of three different approaches to collecting Patient Reported Outcome Measures (PROMs) data using the Prostate Cancer Outcomes Registry-Victoria (PCOR-VIC)

**DOI:** 10.1186/s12913-017-1981-1

**Published:** 2017-01-23

**Authors:** Dewan Md Emdadul Hoque, Fanny Sampurno, Rasa Ruseckaite, Paula Lorgelly, Sue M. Evans

**Affiliations:** 1 Department of Epidemiology and Preventive Medicine (DEPM), School of Public Health and Preventive Medicine, Monash University, The Alfred Centre, Level 6, 99 Commercial Road, Melbourne, VIC 3004 Australia; 2 International Centre for Diarrhoeal Diseases Research in Bangladesh (icddr,b), 68, Shahid Tajuddin Sarani, Mohakhali, Dhaka 1212 Bangladesh; 3 Centre for Health Economics, 15 Innovation Walk, Monash University, Clayton, VIC 3168 Australia; 4 Office of Health Econimics (OHE), Southside, 7th Floor, 105 Victoria Street, London, SW1E 6QT UK

**Keywords:** Data collection methods, Randomized Controlled Trial, Cost-effectiveness, Patient Reported Outcome Measures, Mixed-mode

## Abstract

**Background:**

Patient-reported outcome measures (PROMs) are used by clinical quality registries to assess patients’ perspectives of care outcomes and quality of life. PROMs can be assessed through a self-administered survey or by a third party. Use of mixed mode approaches where PROMs are completed using a single or combination of administration method is emerging. The aim of this study is to identify the most cost-effective efficient approach to collecting PROMs among three modes (telephone, postal service/mail and email) in a population-based clinical quality registry monitoring survivorship after a diagnosis of prostate cancer. This is important to assist the registry in achieving representative PROMs capture using the most cost-effective technique and in developing cost projections for national scale-up.

**Methods/design:**

This study will adopt an equivalence randomised controlled design. Participants are men diagnosed with and/or treated for prostate cancer (PCa) participating in PCOR-VIC and meet the criteria for 12-month follow-up. Participants will be individually randomized to three independent groups: telephone, mail/postal, or email to complete the 26-item Expanded Prostate Cancer Index Composite (EPIC-26) survey. It is estimated each group will have 229 respondents. We will compare the proportion of completed surveys across the three groups.

The economic evaluation will be undertaken from the perspective of the data collection centre and consider all operating costs (personnel, supplies, training, operation and maintenance). Cost data will be captured using an Activity Based Costs method. To estimate the most cost-effective approach, we will calculate incremental cost-effectiveness ratios. A cost projection model will be developed based on most cost-effective approach for nationwide scale-up of the PROMs tool for follow-up of PCa patients in Australia.

**Discussion:**

This study will identify the most cost-effective approach for collecting PROMs from men with PCa, and enable estimation of costs for national implementation of the PCa PROMs survey. The findings will be of interest to other registries embarking on PROMs data collection.

**Trial registration:**

ACTRN12615001369516 (Registered on December 16, 2015)

## Background

Patient reported outcome measures (PROMs) are designed to measure patients’ views of their symptoms, own functional status, treatment satisfaction and health related quality of life in relation to specific disease or conditions [1–4]. PROMs are an important measure of patients’ perspective of care outcomes as they provide insight into the impact of a disease and its treatment on daily lives. PROMs can assist clinicians to work with patients to achieve a level of care that meets their needs; this has been demonstrated to improve patient-provider communication [5].

PROMs may be self-administered or administered by another person (third-party). Instruments used to collect PROMs should be validated for the mode with which they are being administered. Self-administration may include surveys that patients complete on paper or electronically (e.g. via links provided in an email address to an online form or through Applications (apps) that patients can download. Tools administered by another person may include those completed on paper or electronically with assistance or those administered over the telephone [6, 7]. With the increasing number of Internet users, greater opportunities exist for collecting data, through mechanisms such as email and web-based surveys [8, 9]. An emerging trend in health-related survey research is the use of a mixed-mode approach. In the mixed-mode method, individuals may respond using a single or combination of different modes, such as only telephone or mail followed by telephone [10]. Using a mixed-mode approach compensates for the weaknesses of each individual mode at affordable cost [11]. Survey mode, length and content of the survey and incentives will impact the response rates and the cost of data collection [12].

The amount of clinical data being collected is growing exponentially; largely due to computer-based information systems [13]. In Australia, the number of known registries collecting clinical data has risen from 28 in 2006-07 to 37 in 2012 [14]. Clinical quality registries have received increasing attention as a means of improving quality and reducing the cost of health and medical care, through identifying variations in clinical practice and care, and assessing the uptake of effective treatment [15, 16]. A number of clinical quality registries collect PROMs and provide reports on outcomes to hospitals. Examples can be found in trauma [17], joint replacement [18] and renal disease [19]. PROMs are being developed by the American Society of Clinical Oncology to benchmark hospitals in relation to symptoms and functional status following cancer treatment [20].

For any individual research study, the mode of data collection is influenced by time, available resources and the population being targeted [21]. A number of studies have compared response rates using different modes of data collection [22]. A meta-analysis published in 2009 found that email surveys have lower response rates compared with mail surveys (20% vs 53% respectively) suggesting that, despite rapid growth of information technology, mail surveys appear to be superior to email in collecting survey data [22]. High response rates have been obtained when follow up attempts are intense and personalised. For example Steineck et all reported very high response rates of 89 to 99% across multiple time periods by following a regimen which included an introduction letter and a telephone call to establish contact prior to a survey being posted and a “thank you and reminder” card following return of the survey [23, 24]. It is unclear whether such a labour-intensive approach is sustainable at a population level.

Response rates have been found to vary among study populations. Postal surveys with three reminders have shown demonstrably better response rates among general practitioners compared with a telephone survey [25]. A randomised control trial (RCT) of junior medical staff and faculty members comparing electronic and postal surveys found that response rates were similar, but the average response time for electronic surveys was shorter for the residents’ group compared with the faculty group (3.8 days vs 8.4 days, p < 0.001) [26].

A recent meta-analysis by Rutherford et al. [27] investigated whether the mode of PROMs administration introduced bias into the patient reported outcome results. Findings suggested that there was no bias associated with whether PROMs were collected electronically (computer including web, touch screen, hand-held device, video conference, computer assisted telephone interview), via paper self-completion (hard copy) or via assisted completion in clinics or home. The authors of the study recommended further research using experimental designs to measure the mediators of mode effects on data quality, measurement equivalence, reliability of assessment for individuals and the impact of setting and combination of data collection method over time [27].

A cost-effectiveness study by Sinclair et al. (2012) found that postal survey costs were lower compared to both internet and telephone. The cost of a completed response using a personalised postal survey (24.75 Australian Dollars) was slightly higher than the generic postal survey, a generic internet survey and a personalised internet survey cost was almost double of a personalised postal survey and a telephone survey cost was highest among all methods [28]. Another study conducted by the Australian national stroke registry, found that telephone follow up for patient with acute stroke or transient ischemic attack was more expensive but more effective in terms of completion rates than follow-up by postal mail [16].

The Victorian Prostate Cancer Registry (PCOR-VIC) was established in 2009 to monitor treatment and outcomes of men diagnosed with prostate cancer in Victoria. PROMs are collected to assess the impact of prostate cancer diagnosis/treatment on urinary, bowel, hormonal, and sexual function and bother using the EPIC-26 survey [29]. The EPIC-26 survey has been validated for telephone and self-administered survey (paper or online) and is currently only administered by telephone [30]. The response rate has varied over time as modifications have been made to the registry and is currently at 85%. Alternative methods of PROMs administration have not been systematically assessed for their cost-effectiveness and feasibility. Although previous studies demonstrated lower response rates and in some cases increased costs of surveys delivered electronically, these were conducted several years ago and on a different population. As the PCOR-VIC is now contributing to a newly developed Prostate Cancer Registry-Australia and New Zealand, [31] the aim of this project was to assess the most cost-effective approach for collecting PROMs in a prostate cancer population.

The current study protocol describes the design of an equivalence RCT to assess the cost-effectiveness of three different methods of PROMs data collection using the EPIC-26 survey for patients diagnosed with prostate cancer.

**The primary objective** of the trial is to compare the completeness of survey data obtained using the three different data collection approaches for reporting on PROMs.

**The secondary objectives** are to:

- Estimate recurrent costs of data collection using telephone, postal services/mail and electronic mail (email) for PROMs data in PCOR-VIC.
- Compare the cost-effectiveness of the three different methods of data collection.
- Develop a cost projection model to estimate the cost for nation-wide scale-up of administering the PROMs data collection tool in the most efficient setting for follow up of prostate cancer patients in Australia.

## Methods/design

### Setting

Men who are diagnosed with prostate cancer in Victoria, contributing to the PCOR-VIC and who are interviewed by researchers to collect PROMS, will be invited to participate in this study. Since its establishment in 2009 the registry has expanded to 33 hospitals across the state, representing approximately 75% of the Victorian population [32]. Men are eligible for inclusion on the register if they have had a histologically confirmed diagnosis of prostate cancer that is notified to the Victorian Cancer Registry by the hospital.

### Trial design

The study design proposed for this evaluation is an equivalence RCT design. Participants will be individually randomized to one of three independent groups receiving the PROMs instrument by email, post or by telephone. Due to the nature of the intervention, it is not possible to blind the researchers or study participants.

To collect costing data we will use an Activity Based Costing (ABC) method [33] and structured questionnaires to estimate the cost of the operational activities of the three different methods of follow up. The ABC method is useful for understanding key activities of any programs and interventions and allows identification of (i) implementation levels and composition of costs; (ii) variations in how an intervention is implemented over time and associated cost implications; and (iii) resulting costs of increasing coverage of cost-effective data collection methods. This method is flexible, so its resulting estimates can be easily understood and adapted to measure the cost of data collection of the three different methods. Costs that will be considered include personnel cost, cost of supplies (e.g., envelopes, printing etc.), cost of training of data collectors and cost of operation and maintenance (e.g., telephone bill, internet bill, rent etc.) for each of the data collection methods.

### Recruitment of patients

Recruitment of patients to the PCOR-VIC has been previously described [34]. In summary, patients diagnosed in recruiting hospitals and notified by the hospital to the registry are sent details of the registry in an explanatory statement by mail. In the explanatory statement details on what data will be collected from the patient’s medical record and directly from the patient and how a man can opt off the registry if he chooses not to participate are included. The explanatory statement also contains the contact details of both the hospital where the patient was diagnosed and the university conducting the research and hosting the registry. Clinical data are collected on men who do not opt out. A waiver of consent enables clinical details to be collected from men who have died after diagnosis.

Eligible men are contacted by centrally-located university call-centre follow up staff to confirm that clinical data are accurate and up-to-date. For data collection contact is made any time within a window period of 21 days on either side of the anniversary date for data collection (henceforth recorded as the “Anniversary Date”). This is 12 months from the date of the positive biopsy for patients who do not proceed to active treatment or only receive androgen deprivation therapy; or 12 months from the date on which final initial treatment, or course of treatment finished. This is also the date for surgery and low-dose rate (seed) brachytherapy procedure. For radiotherapy and chemotherapy this is the date that the last dose of therapy was provided as well.

### Inclusion criteria

Men will be included in this study if they are eligible and have been included on the PCOR-VIC, are aged >18 years and answer the telephone when contacted by data collectors in the 21 days leading up to and including their Anniversary Date.

### Exclusion criteria

Men will be ineligible for inclusion in the RCT if they opt off the registry, have died in the period between being recruited to the registry and telephoned, do not speak English, are identified as being hearing or mentally impaired when contacted by data collectors to administer the PROMs, have been diagnosed by Transurethral Resection of the Prostate (TURP) and their treating doctor has requested that we do not contact them for follow up, or if they answer the telephone after their Anniversary Date.

## Outcome of interest

### Primary outcome

The primary outcome of interest will be effective successful follow up. Effective successful follow up is defined as providing a response to each of the 26 questions in the survey. Provision exists for patients to record “decline to answer’ on the electronic form and they are advised at the beginning of the phone call that they may chose not to answer if they wish. Responses will still be considered completed if the patient declines to answer.

### Secondary outcomes

Secondary outcome measures include time to complete an effective follow-up, and the number of occasions where answering a question was declined for each mode of survey administration.

## Sample size

Sample size calculations were based on an equivalence study design [35] and the primary outcome measure (completeness of survey responses). Given the current follow up response rates from the PCOR-VIC we estimate that we will achieve a response rate of 90% [32] when PROMs are administered by the telephone follow-up method. The study was designed to evaluate whether the completeness of survey responses in the ‘mail/postal service group’ or ‘email group’ was similar to the ‘telephone’ group. Assuming that the equivalence margin is 10%, we will require 190 respondents per group. We have made an assumption that the 83% of who have internet access will also have an email account [36]. The sample size was adjusted accordingly so the final number of respondents required in each group is 229 giving a total number required n = 687 (Table 1). Our sample size has been calculated to provide a level of significance at 5% with 80% power (2-sided test). The sample size calculation was performed in Stata V13.0 [37].

**Table 1:** Sample size calculation

Indicator	Targeted response rate	Equivalence margin	Sample size (SS) per group	Internet access	SS adjusted for email	Total Sample required
Response rate	90%	10%	190	83%	229	687

## Randomisation process

**Fig. 1:**
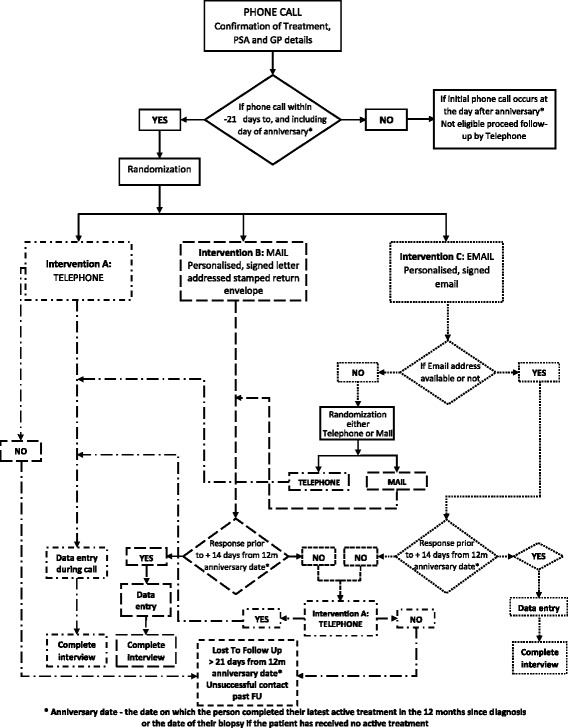
Flow chart showing 3 different follow-up methods

Figure 1 illustrates the different steps for following up patients on the PCOR-VIC registry to complete the PROM (EPIC-26 survey). The first step after data collectors’ telephone to men is to confirm treatment and GP details and obtain the most recent PSA result. If the patient is contacted within the window from 21 days before the “Anniversary Date”, then they will be eligible for randomization. Randomization will be undertaken using random permuted blocks of sizes 3 and 6 [38]. The investigator will have already generated the random blocks and provide those to the data collectors in sealed envelopes. Once the data collector has confirmed the patient’s PSA result, GP details and eligibility for 12-month follow-up then s/he will proceed with selecting a random number from the sealed envelope to assign the method for collection of data by EPIC-26 survey. If a patient has been assigned to the email method but, when the data collector asks for an email address and the patient replies that he does not have one, the data collector will select the next and subsequent envelopes until either the telephone method (Intervention A) or mail/postal methods (Intervention B) is assigned.

### Description of intervention(s)/exposure

An attempt will be made to contact all men who have not opted off the registry and meet the eligibility criteria. The details and procedure followed by data collectors for administering the EPIC-26 survey following randomisation to one of the three approaches is described below and presented in Fig. 1 Flow Chart showing 3 different follow up methods.

**Intervention A**: PROMs data collection by follow-up data collectors over the **telephone**

1. An attempt to contact patients via phone will be made up until 21 days post diagnosis/treatment. Each day a data collector will make one attempt to contact the patient.
2. Patients responding will have PROMS entered directly to the PCOR-VIC web system.
3. Patients will be considered lost to follow up if they do not respond after 21 days from 12 month “Anniversary Date”.

**Intervention B**: Data collection by follow up data collectors by **mail (postal service)**

1. The EPIC-26 survey will be mailed to patients if they are contacted any time from 21 days before, up to and including the “Anniversary Date”. The survey will be accompanied with a personalised, signed letter with instructions and a postage-paid, self-addressed envelope.
2. If the survey is not returned by 14 days post the Anniversary Date, patients will be telephoned as per Intervention A. Data collector will make one attempt each day i.e 14 attempts in total.
3. Patients will be considered lost to follow up if they do not respond after 21 days from 12 month “Anniversary Date”.

**Intervention C**: Data collection by **E-mail** link to online survey

1. If email is not available then the participants will be randomly assigned to either telephone (intervention A) or mail (Intervention B).
2. A Uniform Resource Locator (URL) which is the address of a web page link to the EPIC-26 survey will be emailed to participants if they are contacted any time from 21 days before, up to and including the “Anniversary Date”.
3. If participants do not complete the online survey by 14 days post the Anniversary Date, patients will be telephoned as per Intervention A. Data collector will make one attempt each day i.e 14 attempts in total. Patients will be considered lost to follow-up if they do not respond after 21 days from 12 month “Anniversary Date”.

## Duration of the project

We anticipate that the recruitment process will commence on the 1^st^of February 2016. Data collection will take place between February 2016 and November 2016. Based on current number of patients that become eligible for follow-up each month, we anticipated that it will require approximately 8-9 months to reach our total sample size of 687. An additional 2 months will be required for entering, analysing costing data and for report writing and developing draft manuscripts.

## Data collection, quality and monitoring methods

We will employ quantitative methods of data collection through structured questionnaires. For costing, the ABC [33] method will be adopted. Table 2 describes the data collection methods that will be used to obtain data for each specific objectives. Data will be collected by trained data collectors and supervised by the project manager and study investigators. Data collectors are from an academic institution (Monash University) and are independent of the hospitals. To ensure data quality, validation tools will be built in the web-based system. Routine monitoring meeting will also be held to ensure any problems encountered during data collection and randomization process.

**Table 2:** Description of the specific objectives and methods

	Objectives	Methods
1	To compare the completeness of data across the three data collection approaches.	Total number of completed surveys will be identified in and compared across each of the three PROMS data collection approaches. Both the paper and electronic version provide capacity for patients to decline to answer any question. Provision in the electronic survey exists to alert patients where fields are left blank to prevent missing data issues.
2	To measure the cost of data collection of three different methods: Telephone, Mail (Postal services) and Email for Patient Reported Outcome Measures (PROMs) data in PROC-Vic.	The Activity Based Costing (ABC) method will be followed to estimate the cost of the operational activities of the three different methods of follow up. The costing data will be collected from provider perspective. Table 3: Describes the itemized costs of the 3 different methods.
3	To compare the cost and effectiveness of three different methods of data collection	Cost-effectiveness: Total cost (Personnel, recurrent, supply---)/Number of completed follow-up patients
4	To develop a model to determine the cost for national scale-up of PROMs data collection method with EPIC-26 survey in Australia.	A cost projection model will be developed based on most efficient approach. The model will use epidemiological data and estimated cost parameters. Estimated incidence data on national prostate cancer cases and completed follow up of patients will be forecasted. Using estimated cost data will help to determine the cost for national scale-up of follow up per year. A sensitivity analysis will be conducted with different assumptions e.g. (1) total number of prostate cancer patients; (2) completed follow up surveys and; (3) cost.
5	To provide recommendations for most cost-effective approach for complete follow-up of prostate cancer patient	A comprehensive report with recommendations will be developed.

## Data analysis plan

### Primary and secondary outcomes

For the primary outcome measure (completeness of surveys), we will estimate individual proportions with 95% CI for each arm of the trial. We will then calculate the pooled sample proportions for each pair and the standard error of the difference. Using these measures, we will calculate the Z score test statistics. The p value will be reported for the difference and P < 0.05 will be considered as statistically significant.

For the secondary outcome measures, we will carry out independent sample t-tests to detect the difference.

Analysis will be conducted by intention to treat (ITT) [39] as well as per protocol method [40]. The ITT method will allow us to evaluate the effect of each intervention in a real life situation (i.e. when subjects are randomized to the ‘email group’ but end up in the ‘telephone’ or ‘mail’ methods instead). The per-protocol method will allow us to study the direct effect of each option.

#### Economic analysis

The economic evaluation will consider the costs of personnel resources, supplies, and operating costs as described below. The analysis will not consider development of the PCOR-VIC database, as we consider that this cost will be identical regardless of the data collection modality. However, the additional costs associated with developing the automated email system within the register will be captured.

- Valuing personnel resources
  The value of the research staff contacting patients will be measured in terms of salaries, allowances and benefits received during the study period for their time. At Monash University, all personnel maintain a time sheet for their usual activities. We shall calculate time taken for the data collection processes from time sheets for the period of data collection (February 2016-November 2016). As the level of competence required to undertake all tasks are comparable, we will cost the time using a consistent salary across all three groups.
- Valuing supplies and other recurrent costs
  The values of line items outlined in Table 3 will be included as variable inputs and valued at the price at which they were obtained. Items obtained free of cost will be valued at market price – that is, the price paid if purchased from the local or international market. If price from the international market is used then that price will be changed in Australian dollars using the purchasing power parity (PPP) of Australian dollar with USD in 2015 [41]. The actual expenditure for rent, utilities and supervision during the year will be taken into account [41]. These costs data will be collected from the managers of the data collection facilities using a structured questionnaire.

**Table 3:** Cost data collection of 3 different methods

	Telephone	Mail (Postal service)	E-mail
Personnel time cost (Time to contact and complete patient surveys, as well as administrative tasks such as filing forms, data entry, and checking patient data)	Spread sheet to keep the time of the data collection staff	Spread sheet to keep the time of the data collection staff	Spread sheet to keep the time of the data collection staff
Training cost (Time cost of the trainers and trainee, food costs if any and training materials costs)	Yes	Yes	Yes
Mail (envelope, stamp, mail cost) – printing	No	Yes	No
Email	No	No	No
Telephone costs – all receive initial phone call, plus telephone costs for collection via the telephone.	Yes	Yes	Yes
- Methodology of time allocation of providers
  Data collectors will maintain a spreadsheet to record the time spent for each data collection method. The time will be categorised into direct data collection and non-service professional activities which includes preparatory activities, maintaining and management of records and information, and obtaining supplies.
- *Calculating Total and Average Costs:*
  We will sum personnel costs, cost of supplies, training costs and costs of operation and maintenance to provide a total cost for each of the follow up methods as well as average cost for each constituent items.
- *Estimating cost effectiveness*
  To estimate the most cost-effective approach, incremental cost effectiveness ratios (ICERs) will be estimated. This involves comparing the incremental/additional cost of one approach with the additional outcome achieved by using that approach. Outcome will be measured as completed surveys. The most cost effective approach will be the method which achieves the greatest percentage of complete surveys at the least cost [42]. Scenario analysis will be undertaken to test some of the assumptions in estimating the cost of each approach.

### Cost projection modelling

A cost projection model will be developed based on the most cost-effective approach for collecting completed PROMs surveys. The model will use epidemiological data (Australian prostate cancer incidence data) and the estimated follow-up cost. A sensitivity analysis will be conducted using different follow up rates and cost assumptions.

## Quality assurance

This study will be conducted in accordance with the National Health and Medical Research Council (NHMRC) Code of Responsible Conduct of Research.

## Dissemination of results and publication policy

Results of this study will be disseminated to the scientific community through conferences, seminars presentations and publications in peer-reviewed journals. We will present the findings at the Registry Special Interest Group at Monash University to ensure that the lessons learned in the PCOR-VIC registry are made available to other registry custodians.

## Data storage, access and security arrangements

The PCOR-VIC data are housed on a secure server at Monash University. Security is maintained using encryption of data, a managed and audited protocol for access, training and accreditation of personnel, role-based access and authentication of data. The database storing PROMs and costing data will be password protected and stored on a networked server that is backed up on a daily basis at Monash University.

## Potential risks

As the participants are diagnosed with prostate cancer, they may experience distress when discussing the disease with follow up staff over telephone. There will be no pressure on the participants to divulge any information if they do not feel comfortable to do so. An offer will be made to terminate the interview if the participants exhibit any signs of distress. The interview will only be continued if the participants want to do so. As there will be no physical examination, biomedical tests (invasive/non-invasive) or use of hazardous material, this project is deemed to be a low-risk research activity.

## Discussion

Through this economic analysis we will determine the most cost-effective means of capturing PROMs and completing follow up of prostate cancer patients through the PCOR-VIC using the EPIC-26 survey. Our primary aim therefore is to identify the value of different PROMS collection methods. Value is defined as outcomes relative to costs and encompasses efficiency [43]. The outcome we will be evaluating is response rates and representativeness of the various tools in assessing the quality of life. With knowledge of the cost of each completed survey, we will decide which approach to use as we expand data collection to obtain national coverage. Our primary objective is to assess the response rate across each data collection approach. Surveys will only be considered as completed if all relevant questions have been answered.

This will provide guidance to other registries undertaking follow up surveys of patients and it will also allow us to estimate the cost for collecting PROMs data within the Prostate Cancer Outcome Registry- Australia. Moreover we will be able to understand the quality of PROMs data collected by the three different methods. It may be that one method produces significantly higher rate of missing data or questions which patients decline to answer. We have to weigh the cost of data collection against completeness of surveys to determine the method which provides the greatest value at the end of the study. This decision will be made by the project steering committee and the funder.

## Conclusion

The aim of this study is to provide evidence on which method of PROMs follow up data collection is more cost-effective. Findings of this study will also help us in understanding the cost for national implementation of the prostate cancer quality of life questionnaire in Australia.

## Abbreviations

ABC: Activity Based Costing
EPIC-26: Expanded Prostate Cancer Index Composite
GP: General Practitioner
ICERs: Incremental Cost Effectiveness Ratios
ITT: Intention to Treat
MGS: Monash Graduate Scholarship
MIPRS: Monash International Postgraduate Research Fellowship
NHMRC: National Health and Medical Research Council
PCa: Prostate Cancer
PCOR-VIC: Prostate Cancer Outcome Registry- Victoria
PPP: Purchasing Power Parity
PROMs: Patient Reported Outcome Measures
PSA: Prostate Specific Antigen
RCT: Randomized Controlled Trial
TURP: Transurethral Resection of The Prostate
URL: Uniform Resource Locator
